# Effects of Ethanol on the Toxicokinetics of Methamphetamine in Rabbits

**Published:** 2014

**Authors:** Bing Li, Yujin Wang, Yun Zhang, Meili Liu

**Affiliations:** *Department of Forensic Medicine, Shanxi Medical University, Taiyuan, PR China.*

**Keywords:** Methamphetamine, Amphetamine, Ethanol, Toxicokinetics

## Abstract

In the current study, the effects of ethanol (EtOH) on toxicokinetics of methamphetamine (MA) and its metabolite amphetamine (AP) were investigated. A single dose of MA hydrochloride at 15 mg/Kg was given intragastrically, either alone (MA group; n = 8) or in conjunction with 3 g/Kg EtOH (MA+EtOH group; n = 8) to rabbits. In placebo group, normal saline only was given (placebo group; n = 4). Plasma and urine samples were collected and analyzed by gas chromatography/mass spectrometry (GC/MS) for MA and AP. Toxicokinetic parameters of MA and AP were determined using WinNonlin. Our results showed that toxicokinetic profiles of MA and AP in the two experimental groups were both fitted to an open two-compartment model with first-order kinetics. These were not affected by co-administration of EtOH. However, concomitant intake of EtOH significantly increased MA plasma absorption constant (Ka) and maximum concentration (Cmax). The Ka of MA was increased from 0.679/h ± 0.023/h to 0.964/h ± 0.033/h (P < 0.05, the mean Cmax from 1.408 mg/L ± 0.072 mg/L to 1.676 mg/L ± 0.135 mg/L (P < 0.05), whereas the Tmax was significantly decreased from 1.620 h ± 0.062 h to 1.259h ± 0.033h (P < 0.05). In contrast, no significant difference was observed on MA elimination. Furthermore, the plasma AP area under the curve (AUC0-30 h) increased from 5.281 mg/h/L ± 0.264 mg/h/L to.13.052 mg/h/L ± 0.956 mg/h/L and Cmax increased from 0.315 mg/L ± 0.010 mg/L to 0.423 mg/L ± 0.042 mg/L (P < 0.01). Taken together, co-administration of EtOH with MA significantly accelerated MA absorption and subsequent metabolism to AP, but did not have significant effect on MA elimination.

## Introduction

Methamphetamine (MA) is a psychostimulant, which was FDA approved for treatment of ADHD and obesity. However, methamphetamine has high potential for abuse and addiction. Methamphetamine is rapidly absorbed via gastrointestinal tract and metabolized in liver. The major routes of metabolism are N-demethylation and aromatic hydroxylation. Amphetamine (AP) and *p*-hydroxy methamphetamine are major active metabolites. 

MA abuse is often accompanied with recreational consumption of ethanol (EtOH). It is unclear whether EtOH may change the absorption, distribution, metabolism, and/or excretion of MA. Past studies indicated that ethanol does not seem to significantly affect pharmacokinetic parameters of intravenously administered MA, except an apparent decrease of volume of distribution at the steady state (1, 2). However, the study was limited due to very limited number of human subjects studied. Interestingly, simultaneous administration of AP and EtOH produced greater hypothermia than EtOH alone (3). Additionally, previous studies indicated that plasma concentrations of gamma-hydroxybutyrate or MDMA were increased by co-administration of EtOH (4, 5). 

In the current study, the effects of EtOH on the pharmacokinetics of orally administered MA were characterized in rabbits. Insights from the current study may reveal potential mechanisms underlying drug metabolism and provide valuable reference for blood and urine drug testing.

## Experimental

*Chemicals and analysis softwares*

MA hydrochloride, AP sulfate and propyl adiphenin (SKF_525A,_ IS) were obtained from Chinese National Laboratory of Narcotics, and 1.0 g/L methanol stock solutions were prepared. The derivatization reagent trifluoroacetic anhydride (TFAA, 14.9 g/mL) was purchased from Sigma. All other chemicals and solvents were of analytical grade.

Pharmacokinetic parameters were determined using the WinNonlin Pro computer program, standard edition (Pharsight Co., USA).

SPSS11.5 statistical software was purchased from SPSS Co. (USA).

*Animals*

Twenty white male rabbits, each weighing 2.0 Kg ± 0.1 Kg, were bred by the Laboratory Animal Center of Hebei Medical University. Sixteen rabbits were randomly divided into two experimental groups, receiving a single, oral dose of MA hydrochloride alone (MA group) or with EtOH (MA + EtOH group). The remaining four rabbits received normal saline as the placebo-controlled group. The handling and use of animals were in accordance to the institutional guidelines and all experiments were carried out in accordance with current and ethical guidelines for the care and use of laboratory animals.

*Administration protocol*

After overnight fasting, a single dose of 15 mg/Kg body weight of MA hydrochloride solution was administered intragastrically in the MA group or with 3 g/Kg EtOH in the MA + EtOH group. The same volume of normal saline was administered intragastrically to the placebo-controlled group. Changes in vital signs including ECG, blood pressure and respiration rate were monitored and recorded for 3 hours by “BL-Physiological Function Experimental System (Chengdu Taimeng Co.，Ltd, China).

*Experiments*

Blood samples were collected before administration and at 0.5, 1, 1.5, 2, 2.5, 3, 5, 8, 12, 16, 20, 24 and 30 h after drug administration. Likewise, urine samples were collected at 0.5, 1, 2, 5, 8, 12, 16, 20, 24 and 30 h. The plasma and urine samples were stored at -20 °C until analysis.

One milliliter of plasma and urine from each animal was diluted with 2 mL boric acid buffer solution and subsequently mixed with 200 ng of the internal standard SKF_525A. _The samples were processed by liquid-liquid extraction. To each sample, 5 mL diethyl ether was added and vortexed for 5 min. After centrifugation (3000 r/min) for 10 min, the organic layer was transferred into a clean test tube and all these extraction steps were repeated once again. After a drop of acidic methanol was added, the organic layer was evaporated dry by water bath at 40 ºC. MA and AP in the residue were subjected to acylation derivatization by 25 µL TFAA and 25 µL ethyl acetate in a microwave oven for 2 min, and evaporated dry again by a stream of nitrogen. The residue was reconstituted with methanol. Aliquots of 1.0 µL were injected into the GC system. 

*Analytical methods*

A GC-MS (TRACE GC&DSQ Model, Thermo Finnigan, USA) system equipped with a splitless injection port, and a capillary column DB-5MS (30 m×0.25 mm×0.25 μm) was used for analysis. Carrier gas was high-purity helium (99.999%) at a constant flow of 1.0 mL/min. The temperature for GC was raised from 70 ºC to 200 ºC at 20 ºC/min, and then to 280 ºC at 30 ºC/min, remaining at 280 ºC for 1min. The temperatures for the injector port, the transfer line heater and the ion source (EI) were all set at 250 ºC. The electron energy was 70 eV. At the automatic tuning mode, the multiplier voltage was 1557 V and the current intensity was 100 µA. The mass spectrometer was operating in the full scan mode and the scanning range of 50 amu to 350 amu for qualitative analysis. Quantitative analysis employed the selected ion monitoring (SIM) mode at different time windows: AP-TFAA (m/z: 91,118,140), from 4.0 min to 5.2 min; MA-TFAA (m/z: 91,110,154), from 5.2 min to 6.3 min; SKF_525A_ (m/z: 86, 99), from 9.5 min to 10.2 min. The dynode was shut off between 6.3 min and 9.5 min.

*Method validation*

Plasma and urine samples from controlled group were used as negative controlls. The calibration curves were constructed using linear regression analysis based on the concentration of drugs and the ratio of the peak area of MA or the amphetamine metabolite to that of the internal standard SKF525A. 

*Pharmacokinetic parameters and statistical analysis*

Pharmacokinetic parameters of each drug, including C_max_ (peak plasma concentration), *t*_max_ (time to peak concentration), *t*_1/2_ (terminal half-life), AUC_0-30h_ (area under the plasma concentration- time curve), CL (total body clearance) and V/F (apparent volume of distribution) for MA and its main metabolite AP, were calculated based on the compartment model using the WinNonlin Pro computer program. Statistical analysis was carried out using SPSS 11.5.

The data were analyzed by mean ± standard deviation and the level of statistical significance was P < 0.05. 

## Results

*Ac* *curacy and precision*

Concentrations of MA and AP were determined by using liquid–liquid extraction and gas chromatography–mass spectrometry (GC-MS) The lower limits of detection (LOD) and lower limits of quantification (LOQ) for plasma and urine are presented in Table 1. The correlation coefficients between y and x-values are ≥ 0.999 calculated by the weighted least-squares method. The accuracy and precision of MA and AP in plasma and urine are presented in Table 2. Intra-day and inter-day imprecision were ≤ 8.51% and 7.76%, respectively. 

**Table 1:** Calibration equation and limits of detection of MA and AP in plasma and urine

	**Drugs**	**Calibration equation**	**LOD** **(mg/L)**	**LOQ (mg/L)**	**Correlation Coefficients**
**Plasma**	AP	Y=2.6028×10^-2^+2.425×10^-4^X	0.005	0.01	0.9996
	MA	Y=4.0286×10^-2^+4.602×10^-4^X	0.005	0.01	0.9994
**Urine**	AP	Y=2.414×10^-2^+1.535×10^-4^X	0.010	0.05	0.9998
	MA	Y=2.590×10^-2^+1.044×10^-4^X	0.010	0.05	0.9996

**Table 2:** Accuracy and precision of MA and AP concentration measurement in plasma and urine (n=8).

**Samples**	**Drugs**	**Add in** **(mg/L)**	**Determined** **(mg/L)**	**Recovery** **(％)**	**Intraday RSD(％)**	**Interday RSD(％)**
**Plasma**	AP	0.02	0.020±0.004	98.55±2.94	5.35	7.23
		0.1	0.103±0.004	97.43±3.91	3.62	4.46
		1.0	0.990±0.019	97.29±5.22	4.73	5.22
	MA	0.02	0.019±0.004	97.15±1.56	7.81	4.37
		0.1	0.098± 0.002	98.38±3.67	3.27	6.02
		1.0	1.032±0.029	97.75±4.43	4.05	3.87
**Urine**	AP	0.5	0.502±0.005	98.31±1.92	8.51	5.91
		5.0	5.017±0.017	97.94±2.33	3.93	5.68
		20.0	20.014±0.021	98.44±3.01	3.55	6.55
	MA	0.5	0.493±0.009	97.46±2.18	5.24	3.43
		5.0	4.999±0.023	98.40±1.57	6.04	7.76
		20.0	19.998±0.012	98.72±2.09	4.21	4.62

*MA and AP in plasma*

**Figure 1:**
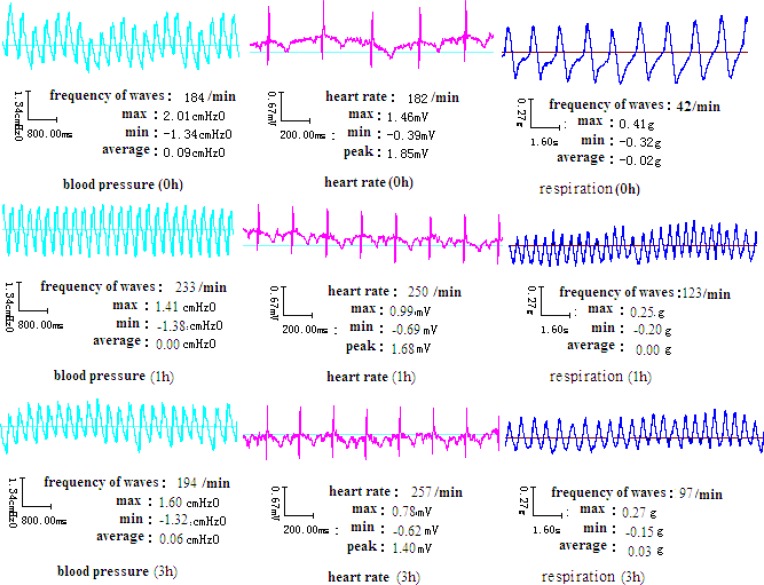
Mean concentration-time curve of plasma MA after administration of MA alone or co-administration of MA and EtOH (n=8).

**Figure 2:**
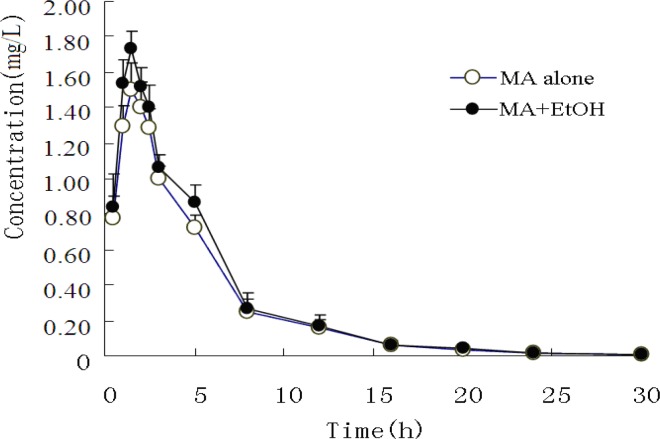
Mean concentration-time curve of plasma AP after administration of MA alone or co-administration of MA and EtOH (n=8).

The mean plasma concentrations of MA and its metabolite AP in both experimental groups are presented in Figure 1 and Figure 2. 

**Figure3:**
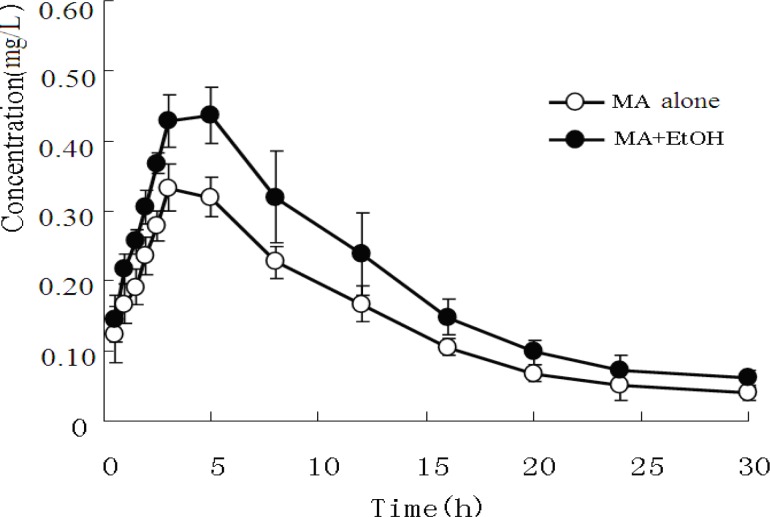
Ratios of AP to MA in rabbit plasma

The pharmacokinetic profiles of MA fitted to an open two-compartment model with first-order kinetics. Co-administration with EtOH did not change its pharmacokinetic model. The pharmacokinetic parameters for MA and AP are listed in Table 3 and Table 4. The ratios of AP to MA in rabbit plasma are shown in Figure 3.

**Table 3:** Toxicokinetic parameters of MA after administration of MA alone or co-administration of MA and EtOH (n=8).

**parameters**		**MA group **	**MA+EtOH group **
Ka* V/F(c) t_1/2__α_ t_1/2__β_ t_1/2ka*_ K_21_ K_10_ K_12_ AUC_0-30h_ CL T_max_* C_max_**	(/h ) (L/kg) (h) (h) (h) (/h ) (/h ) (/h ) (mg/h/L) (L/h/kg) (h) (mg/L)	0.679±0.023 8.399±0.456 0.982±0.102 5.823±0.369 1.021±0.021 0.233±0.011 0.435±0.028 0.182±0.027 8.212±1.039 3.653±0.125 1.620±0.062 1.408±0.072	0.964±0.033 8.027±1.000 0.917±0.026 4.905±0.987 0.719±0.023 0.324±0.015 0.414±0.612 0.195±0.003 9.022±0.069 3.325±0.152 1.259±0.033 1.676±0.135

*P﹤0.05, **P﹤0.01

**Table 4:** Toxicokinetic parameters of AP after administration of MA alone or co-administration of MA and EtOH (n=8).

**parameters**		**MA group **	**MA+EtOH group **
Ka V/F(c) ** t_1/2__α_ t_1/2__β_** t_1/2ka_ K_21_ K_10_** K_12_ AUC_0-30h_** CL* T_max_ C_max_*	(/h ) (L/kg) (h) (h) (h) (/h ) (/h ) (/h ) (mg/h/L) (L/h/kg) (h) (mg/L)	0.266±0.033 37.774±1.538 2.637±0.024 6.919±0.854 2.609±0.222 0.061±0.021 0.150±0.032 0.086±0.023 5.281±0.264 5.681±0.301 4.151±0.411 0.315±0.010	0.259±0.023 29.362±1.814 3.143±0.051 9.978±1.258 2.673±0.497 0.021±0.010 0.078±0.009 0.128±0.015 13.052±0.956 2.298±0.312 4.421±0.744 0.423±0.042

﹡P﹤0.05,** P﹤0.01

When MA was administered intragastrically to rabbits, it was rapidly absorbed, with a short T_max_ of 1.62 h. Concomitant intake of EtOH caused a significant increase in the plasma MA absorption constant (K_a_) and maximum concentration (C_max_). The mean MA K_a_ values increased from 0.679 h ± 0.023 h to 0.964 h ± 0.033 h (P < 0.05), and the mean C_max _from 1.408 mg/L ± 0.072 mg/L to 1.676 mg/L ± 0.135 mg/L (P < 0.05), whereas plasma MA T_max_ appeared to be significantly decreased from 1.620 h ± 0.062 h to 1.259 h ± 0.033 h (P < 0.05). Finally, no significant difference was observed on MA elimination. 

Furthermore, when EtOH was co-administered, the plasma AP area under the curve (AUC_0-30h_) and C_max_ increased from 5.281 mg/h/L ± 0.264 mg/h/L to.13.052 mg/h/L ± 0.956 mg/h/L and from 0.315 mg/L ± 0.010 mg/L to. 0.423 mg/L ± 0.042 mg/L, respectively (P < 0.01). 

*MA and AP in urine*

The mean concentrations of MA and AP in urine are listed in Table 3. In the MA only group, after administration of MA, the mean ratio of AP to MA in urine rose from 0.022 to 0.088 over the experimental time course. In the MA + EtOH group, the ratio rose from 0.020 to 0.084. There was no statistic difference between the two groups. The ratio of metabolic product versus its parent drug in urine often provides some clues regarding whether the parent drug is given solely or together with its metabolic products (6).The fact that AP/MA ≤0.1 indicates that the rabbits were given MA only, not together with AP. 

*Changes of vital signs in rabbits*

**Figure 4:**
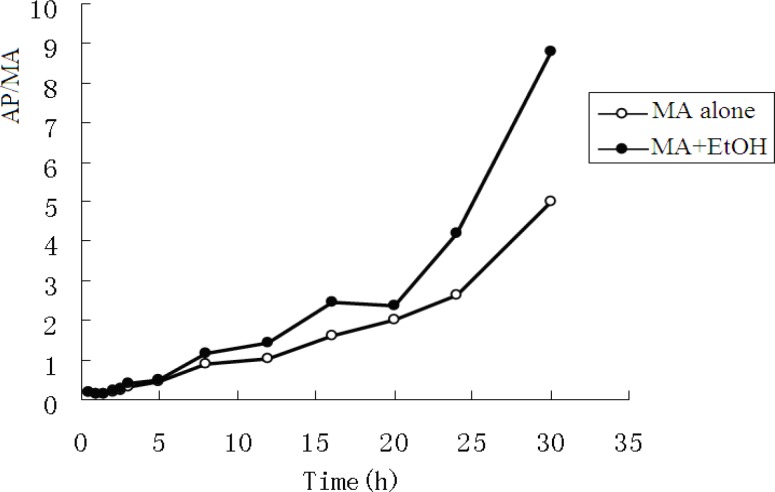
Vital signs of rabbits after intragastric administration of MA

20 min to 30 min after MA intake, the rabbits began to show significant behavioral changes, including nasal ala flap, tongue sticking out, teeth grinding and intermittent limb tremors. The blood pressure of rabbits was elevated initially and then decreased to normal level after MA plasma concentration dropped to 75% of its peak level (Figure 4). The respiration rate reached peak at about 1 h. The heart rate remained to be elevated for more than 3 h. 

## Discussion

*T* *oxic symptoms*

Previous research has demonstrated that a single intraperitoneal administration of MA (10 mg/Kg) induced stereotypic behavior such as continuous sniffing, circling and nail-biting in male ICR mice (7). These behaviors reached a plateau level at 20 min and persisted for at least 1 h after the injection. Similarly, our data showed that a single intragastric administration of MA (15 mg/Kg, *i.g*.) induced changes in blood pressure, respiration and heart rate of rabbits in 20 to 30 min, lasting at least for 1 h. 

*Toxicokinetic profiles of MA in rabbits*

Our results showed that the apparent volume of distribution (Vd) of both MA and its metabolite AP exceeded the total body fluid in rabbits (1.2 L or 0.6 L/Kg), indicating that MA readily accumulated in tissues and the MA content was lower in blood than that in tissues.

Previously, Schepers *et al*. investigated the pharmacokinetics of MA hydrochloride after oral administration of sustained-release tablets to human at a dose of 10 mg and 20 mg (8). They observed that the concentration-time curve was fitted to an open one-compartment model with first-order kinetics followed by a lag time. However, we observed that the concentration-time curve of MA was fitted to an open two-compartment model with first-order kinetics. The discrepancy is likely secondary to different pharmaceutical formulation and dosage, although species differences between rabbits and humans may play a role. 

*Effects of EtOH on the toxicokinetics of MA*

Two previous studies investigated the interactions of MA and EtOH by intravenous administration of MA and oral administration of EtOH to humans, and concluded that EtOH decreased the Vd of MA at steady state and the pharmacokinetics of intravenously administered methamphetamine was not markedly affected by ethanol (1, 2). However, in our experiment, there was no statistic difference in the Vd between the MA and MA + EtOH groups. This is likely attributable to the distinct routes of administration. When MA was administered by vein to the rabbits, MA exhibited significantly slower translocation from blood to tissues. When administered through stomach feeding; first-pass effect, numerous gastrointestinal factors, as well as individual and species differences likely played a concerted regulatory role. MA was initially precluded from blood circulation until absorbed by the gastrointestinal tract and after first-pass effect. As a result, plasma concentration of MA was reduced, and it became more difficult to monitor the pattern of MA distribution from blood to tissues. Accordingly, no statistic difference in the Vd of the central compartment was detected between the two groups.

*Metabolism and excretion of MA in rabbits*

Metabolism and excretion of MA in rabbits were somewhat different from those in humans. AP is a major active metabolite of MA both in humans and in rabbits. However, in humans, AP is further metabolized into hydroxyamphetamine，4-oxedrine and norephedrine via hydroxylation. In the current study, the hydroxylation products of AP were not observed. Previous studies showed that in healthy adults receiving 80 mg of sustained-release MA daily for several days, AP arising from the metabolism of MA in human reached peak levels after about 12 hours. Hence, AP formed from MA is unlikely to contribute significantly to clinical effects (9). However, our results （Figure 2 and Table 5） revealed rapid metabolism and excretion of MA. The exact mechanism underlying such differences remains to be elucidated.

**Table 5:** Mean concentration of MA and AP in urine after administration of MA alone or co-administration of MA and EtOH (n=8).

**T(h)**	**MA group**			**MA+EtOH group**		
	**MA(** **m** **g/L)**	**AP(** **m** **g /L)**	**AP/ MA**	**MA(** **m** **g /L)**	**AP** **(** **m** **g /L)**	**AP/ MA**
0.5	7.996±0.851	0.200±0.019	0.025	9.448±1.113	0.198±0.023	0.021
1	16.626±1.064	0.366±0.030	0.022	18.682±1.568	0.374±0.033	0.020
2	39.865±1.798	1.076±0.057	0.027	40.001±4.541	1.400±0.065	0.035
5	26.722±0.901	1.015±0.076	0.038	26.006±4.332	0.806±0.054	0.031
8	17.121±1.573	0.942±0.039	0.055	16.988±3.215	0.747±0.039	0.044
12	14.116±1.865	0.819±0.085	0.058	12.652±3.584	0.860±0.102	0.068
16	12.863±2.066	0.886±0.052	0.069	9.361±3.210	0.608±0.069	0.065
20	9.003±0.660	0.657±0.032	0.073	9.030±0.698	0.713±0.034	0.079
24	7.123±0.681	0.627±0.046	0.088	6.921±1.062	0.547±0.025	0.079
30	7.884±0.625	0.639±0.036	0.081	6.654±0.012	0.559±0.068	0.084

## Conclusion

Our results indicated that oral administration of MA (15 mg/Kg, *i.g*.) can cause toxic symptoms in rabbits, and EtOH accelerated absorption and subsequent metabolism of MA, but not elimination. Since EtOH appears to increase the toxicity of MA, we suggest that the interaction between EtOH and MA should be considered in MA and EtOH mixed abuse.
